# Multiple S-Layer Proteins of *Brevibacillus laterosporus* as Virulence Factors against Insects

**DOI:** 10.3390/ijms24021781

**Published:** 2023-01-16

**Authors:** Luca Ruiu

**Affiliations:** Dipartimento di Agraria, University of Sassari, Viale Italia 39/A, 07100 Sassari, Italy; lucaruiu@uniss.it

**Keywords:** entomopathogen, biopesticide, biological control, pests, mode of action, pathogenesis

## Abstract

S-layers are involved in the adaptation of bacteria to the outside environment and in pathogenesis, often representing special virulence factors. Vegetative cells of the entomopathogenic bacterium *Brevibacillus laterosporus* are characterized by an overproduction of extracellular surface layers that are released in the medium during growth. The purpose of this study was to characterize cell wall proteins of this bacterium and to investigate their involvement in pathogenesis. Electron microscopy observations documented the presence of multiple S-layers, including an outermost (OW) and a middle (MW) layer, in addition to the peptidoglycan layer covering the plasma membrane. After identifying these proteins (OWP and MWP) by mass spectrometry analyses, and determining their gene sequences, the cell wall multilayer-released fraction was successfully isolated and used in insect bioassays alone and in combination with bacterial spores. This study confirmed a central role of spores in bacterial pathogenicity to insects but also detected a significant virulence associated with fractions containing released cell wall multilayer proteins. Taken together, S-layer proteins appear to be part of the toxins and virulence factors complex of this microbial control agent of invertebrate pests.

## 1. Introduction

Bacterial cells are commonly surrounded by proteinaceous envelops, known as S-layers, that have both structural and biological functions deriving from adaptations to the environment in which they evolved and in which they interact with living and non-living forms [1]. Multiple functions have been attributed to these ordered two-dimensional protein layers, including protection to the plasma membrane, recognition and attachment to external surfaces, enzyme scaffolding, and host interaction, including the adhesion to epithelial cells, which would play an important role in the case of probiotic species [2]. The S-layer of different bacterial species was also found to be involved in pathogenesis, as virulence factors, and in the stimulation of the host’s immune system [3]. Consistently, S-layer genes of some strains of insect pathogenic bacteria, such as the well-known *Bacillus thuringiensis* Berliner, were discovered to encode for parasporal body proteins, which has raised interest for their application potential [4]. The direct involvement of S-layer proteins of different entomopathogenic bacteria in the insecticidal action has also been reported [5,6]. A particular case of extracellular surface-layer protein overproduction is represented by bacteria in the *Brevibacillus* genus [7]. These include the spore-former *Brevibacillus laterosporus* Laubach, whose entomopathogenic properties have been studied against a variety of insects in different orders, including coleopteran, lepidopteran, and dipteran pests [8]. Early studies with this bacterial species on mosquito larvae revealed variable degrees of insecticidal activity associated with different fractions of the bacterium, such as vegetative cells, spores, or liquid culture supernatant [9,10]. These findings were confirmed by more recent investigations in which an increasing insecticidal efficacy against Diptera was, in the following order, related to cultured young vegetative cells (at log phase), old vegetative cells (at stationary phase), and spores (and the sporangia containing them) [11,12]. This led to the identification of virulence factors in the spores, such as the surface proteins of the spore-coat and the canoe-shaped parasporal body firmly attached to it [12]. However, this discovery could not explain the full pathogenicity observed, which supported the hypothesis of a more complex mechanism of action, involving a plethora of substances [13]. Accordingly, genomic sequencing of different *B. laterosporus* strains revealed several putative toxins and virulence factors involved in insecticidal activity against various target pests, even if evidence of their actual involvement in the pathogenic process would require specific experiments [14].

Transcriptomic studies have highlighted the expression during pathogenesis of genes encoding for chitinases, proteases, bacillolysin, serine proteinase, and the insecticidal toxin MTX, homologous to Cry75Aa (corresponding to Mpp75Aa based on the new nomenclature for pesticidal proteins of bacteria) [13,15].

While many aspects of the mode of action still need to be clarified, with respect to the observed action of vegetative cells, the possible involvement of the abundant wall protein multilayer of *B. laterosporus* remains unexplored.

The purpose of this study was to characterize the cell wall multilayer proteins of an insecticidal *B. laterosporus* strain and to investigate their possible involvement in the pathogenic action. This involved ultrastructural observations and proteomic analyses on S-layer proteins and the assessment of their implication in the insecticidal action, employing susceptible house fly adults as a study model.

## 2. Results

### 2.1. Multilayer Cell Wall Structure and Shedding

Pursuing the goal of characterizing the S-layer of *B. laterosporus*, specific observations of this bacterium at progressive stages of growth were conducted using transmission electron microscopy (TEM).

**Figure 1:**
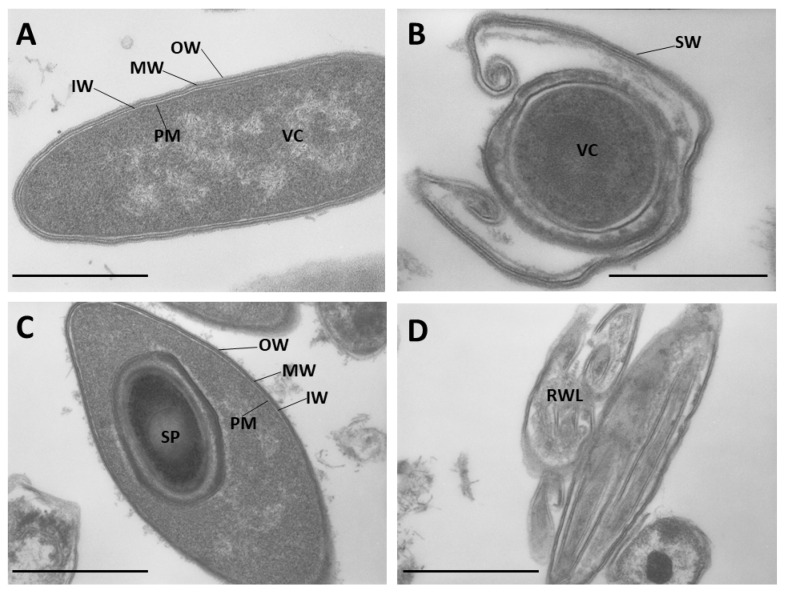
*Brevibacillus laterosporus* transmission electron microscopy (TEM) micrographs showing different bacterial structures. Vegetative cell (**A**). Cell wall shedding (**B**). Sporangium (**C**). Released cell wall multilayers (**D**). VC: vegetative cell; PM: plasma membrane; OW: outer wall layer; MW: middle wall layer; IW: inner wall layer; SW: shedding wall; SP: spore; RWL: released cell wall multilayers. Bar: 500 nm.

Vegetative cells at the logarithm phase showed a typical three-layer cell wall that comprises, proceeding from the outside to the inside, an outermost (OW), a middle (MW), and an inner (IW) layer covering the plasma membrane (Figure 1A), with the middle being the thickest and the inner corresponding to the peptidoglycan layer. Significant cell wall alterations were noted in the late stationary phase, in which the superimposed outer layers started to shed. In several cases, the cell wall unwrapping was more pronounced with the release of wall residues, and additional external multilayers were also observed (Figure 1B). As a result, in sporulated cultures, harvested by centrifugation, sporangia with the cell wall intact and wall remnants possibly resulting from both unwrapping of vegetative cells and sporangia lysis could be observed at the same time (Figure 1C). A fraction of released cell wall multilayers (RWL) was successfully separated by differential centrifugation from cells and spores (Figure 1D). Since this fraction was enriched with S-layer proteins, it was directly employed in bioassays after protein quantification, to evaluate its possible insecticidal effects.

### 2.2. Isolation and Identification of Cell Wall Proteins

The main hypothesis in this study is that S-layer proteins might be involved in the insect pathogenic action, so proteomic analyses were conducted to identify and characterize these proteins in the bacterial fractions used in bioassays.

**Figure 2:**
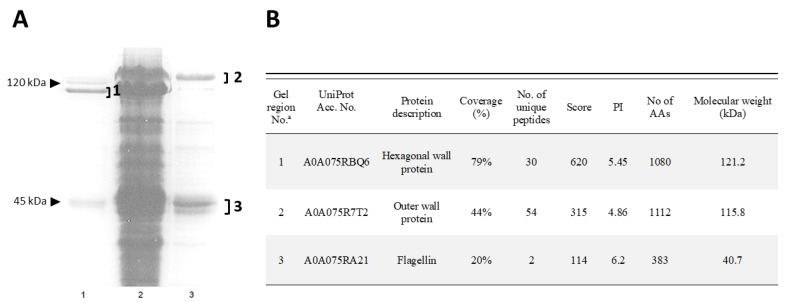
*Brevibacillus laterosporus* vegetative cell major proteins. (**A**) Protein pattern of whole vegetative cells (Lane 2) and surface-layer proteins extracted with guanidine hydrochloride (Lane 1) and EDTA (Lane 3) methods. (**B**) Major protein band identification by mass spectrometry. “a” indicate gel regions excised from 1-DE gel for Mass Spectrometry analyses numbers; “1, 2, and 3” indicate the specific gel region excised from the gel.

The 1-DE profile of vegetative cell proteins, extracted by sodium dodecyl sulfate (SDS) and β-mercaptoethanol from cells at the log-phase of growth, showed major bands in correspondence to a molecular weight of around 120 kDa, which were assumed to be the surface-layer proteins, according to previous knowledge on other *Brevibacillus brevis* group species [7]. Therefore, two specific methods of extraction, respectively employing guanidine hydrochloride or EDTA, were applied. This led to obtaining two extracts, one enriched with outer (OW) and the other with middle (hexagonal, HW) wall proteins, as confirmed by liquid chromatography with tandem mass spectrometry (LC-MS/MS) analysis of the main bands resolved by 10% sodium dodecyl sulphate—polyacrylamide gel electrophoresis (SDS-PAGE) (Figure 2A,B). The protein profile of these fractions also highlighted another band with an apparent molecular weight of around 40 kDa, corresponding to flagellin, which is a key component of the flagella with which this bacterial species is equipped.

DNA sequences of genes encoding for HWP and OWP proteins were deposited in the GenBank database (National Center for Biotechnology Information, Bethesda, Maryland) under accession numbers OP948015 (*hwp* gene) and OP948016 (*owp* gene).

According to the sequences of homologous genes of the *B. laterosporus* genomes available in the NCBI database, these genes appear to be highly conserved in *B. laterosporus*.

**Figure 3:**
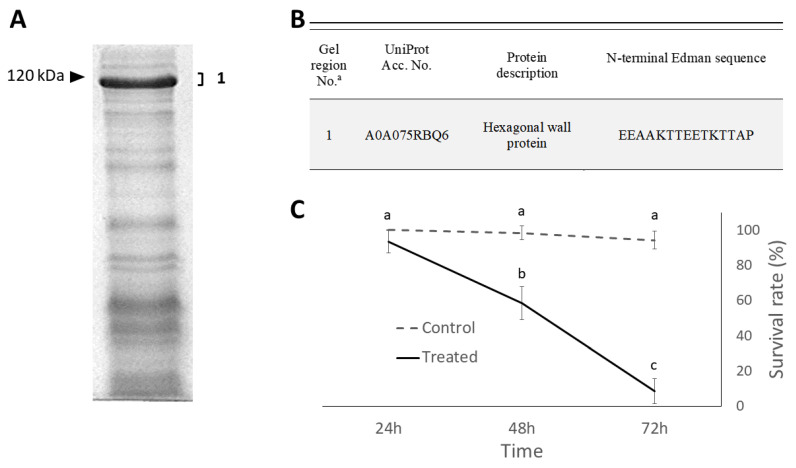
Protein profile and virulence of the whole sporulated culture of *B. laterosporus*. (**A**) SDS-PAGE showing a major band just below 120 kDa. (**B**) Identification of the main protein band by N-terminal sequencing. (**C**) Over time survival (mean ± SD) of house fly adults treated with the whole sporulated culture. Different letters indicate significantly different means (ANOVA proc. mixed, least square means (LSMEANS) adjust = Tukey, *p* < 0.001). “a” indicate gel regions excised from 1-DE gel for Mass Spectrometry analyses numbers; “1, 2, and 3” indicate the specific gel region excised from the gel.

In relation to the release of abundant extracellular proteins in the liquid culture medium, observed in several *Brevibacillus* species, this preliminary characterization work on vegetative cells was followed by analyses aimed at identifying the same proteins in the bacterial fractions showing the highest insecticidal activity. Consistently, the whole sporulated culture collected by centrifugation and found to be highly active against insects is mainly characterized by the presence of free spores and released cell wall multilayers. The 1-DE protein pattern of this fraction clearly showed a major band with an apparent molecular weight just below 120 kDa (Figure 3A). While spores are well-known insecticidal components of this fraction, the protein virulence factors they carry do not include proteins with such molecular weight, which stimulated interest in the direct identification of this major protein band. According to the N-terminal Edman sequencing, this band was identified as the hexagonal (middle) cell wall (HWP) protein (Figure 3B).

While this result indirectly supported the possible involvement of this cell wall protein in insecticidal action, to strengthen this hypothesis, the released cell wall multilayers fraction (RWL), enriched with HWP and OWP proteins, was directly employed in the insect bioassays described below.

### 2.3. Insecticidal Effects

Time-course survival of house flies treated with the whole sporulated culture of *B. laterosporus* administered at a concentration of 10^9^ spores/mL was significantly affected by treatment (F_1,66_ = 942.68; *p* < 0.001), time (F_2,66_ = 336.37; *p* < 0.001), and the interaction between these factors (F_2,66_ = 254.49; *p* < 0.001). A mortality rate of more than 90% was achieved in 72 h (Figure 3C). This result proved the insecticidal efficacy of the sporulated culture containing spores, but also the middle cell wall protein (HWP) as a major protein. Subsequent experiments were therefore aimed at comparing the efficacy of bacterial preparations that did or did not contain this protein.

**Figure 4:**
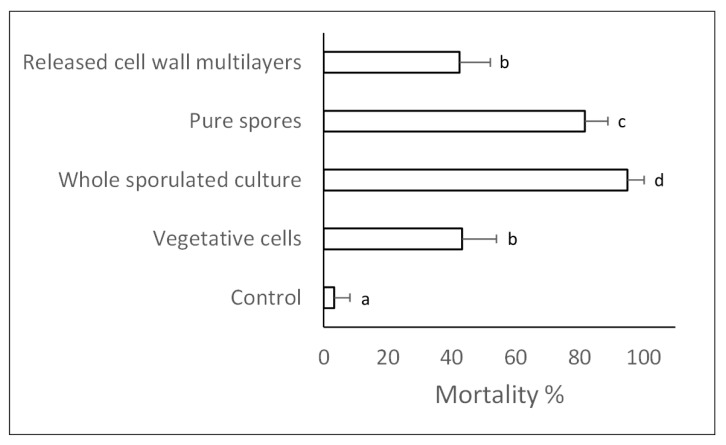
Mortality (mean ± SD) of house flies treated with different bacterial preparations at the normalized concentration of 10^9^ cells/spores per mL. Different letters beside the bars indicate significantly different means (ANOVA, followed by least significant difference (LSD) test, *p* < 0.001).

Significant insecticidal effects were determined by all bacterial stages of growth and fractions assayed, albeit with different effectiveness (F_4,55_ = 253.21; *p* < 0.001). The highest fly mortality exposed to the different bacterial preparations at the normalized concentration of 10^9^ cells/spore per mL was found to be associated with the whole sporulated culture (95%), followed by pure spores (82%) (Figure 4). Vegetative cells and the released cell wall multilayer fraction were also found to have insecticidal activity, albeit weaker (<40%) compared to spore-containing preparations. While these experiments confirmed a central role of spores in bacterial pathogenicity to insects, a significant virulence was also found to be associated with fractions containing released cell wall proteins.

**Figure 5:**
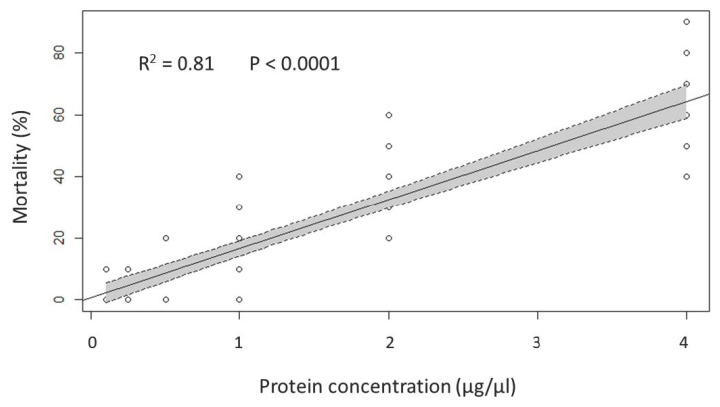
Relationship between fly mortality and protein concentration of the released cell wall multilayer (RWL) fraction of *B. laterosporus* (linear regression plot with shaded areas representing 95% confidence intervals).

The bioinsecticidal activity of the RWL fraction containing cell wall proteins was observed to be concentration-dependent, with a significant correlation between the mortality rate and the protein concentration (adjusted R^2^ = 0.8149, F = 313.5, *p* < 0.0001) (Figure 5).

These results support a role of the cell wall proteins in the complex *B. laterosporus* pathogenic process against insects.

## 3. Discussion

S-layer proteins represent a bacterial tool to interact with the outside environment, and in the case of pathogenic species, they have often been found to play a significant role in the interaction with the host. Such interaction may lead to an increased ability to adhere to the host surfaces or to a more direct participation as virulence factors in the initial pathogenic process [1]. Such properties of vegetative cell-surface layers have occasionally been reported for entomopathogens, such as the well-known *B. thuringiensis* [4,5]. Interestingly, the insect pathogenic bacterium *B. laterosporus*, similarly to other *B. brevis* species group members, is characterized by an over-production of these proteins, which was supposed to be related to an improved capacity to interact with the surrounding environment [7]. Accordingly, our observations confirmed the presence of multiple S-layers, including an outermost (OW) and a middle (MW) layer, in addition to the peptidoglycan layer covering the plasma membrane [16,17]. While such superficial structure is shared with other *Brevibacillus* species, *B. laterosporus* was observed to have its own proteins. On the other hand, the sequence of genes encoding for these proteins appears to be well-conserved in this bacterial species, which would align with a distinct evolutionary path, possibly associated with its entomopathogenic properties. Our study suggests an implication of these proteins in the interaction with the host, ultimately improving *B. laterosporus* virulence overall.

Early studies on *B. laterosporus* had already highlighted the insecticidal potential of vegetative cells toward insects. Specific experiments with *Culex quinquefasciatus* Say (Diptera: Culicidae) and *Aedes aegypti* L. (Diptera: Culicidae) larvae had also shown that the biocidal properties were attributable to the “matter” of which the cells were made, rather than to the living cells themselves [9,10]. In addition, a stronger insecticidal power is normally associated with older rather than younger vegetative cells, suggesting that the aging process of the cell corresponds to an increase in its insecticidal potential, while reducing its replication capacity [12]. At this stage of bacterial growth, we observed conspicuous unwrapping of the outermost layers of the cell wall multilayer with their release into the culture medium, from which, if not specifically removed, they are collected by centrifugation together with the vegetative cells. Accordingly, harvesting spores by centrifugation from a liquid culture at a more advanced growth stage (sporulation) will equally be characterized by the presence of the remaining fractions of the protein S-layer released in the medium. This was confirmed by our electron microscopy observations and, consistently, major protein bands in the sporulated culture of the bacterium were represented by S-layer proteins. In analogy to previous studies with *B. brevis* [7], we successfully isolated and separated form-free spores by differential centrifugation, the cell wall multilayer released fraction (RWL), highlighting its moderate but significant concentration-dependent insecticidal effect when administered to insects as a standalone active substance. Former experiments employing the same *B. laterosporus* strain, UNISS 18, highlighted a prominent role of spores in the insecticidal action, while the potential of vegetative cells was considered of minor importance [12]. While this concept was confirmed in this study, an increased biocidal effect of the whole sporulated culture with respect to pure spores was also detected. The sporulated culture is a mixture of lysed sporangia remnants and released cell wall multilayers, in addition to free spores. Hence, the protein profile of this fraction is characterized by major proteins with a molecular weight of around 120 kDa, which according to LC-MS and N-terminal sequencing analyses corresponds to S-layer proteins. Therefore, their presence in the sporulated culture supports a role for these proteins in increasing the insecticidal potential of the free spores alone, thus explaining the increased insecticidal activity associated with this fraction. 

The implication of surface-layer proteins of *B. laterosporus* vegetative cells in the insecticidal action is reminiscent of the already reported involvement of proteins detected on the surface of the spore-coat and canoe-shaped parasporal body complex typical of this bacterium [12]. After all, this aligns with a more general strategy of bacteria to evolve their surface with adaptations, improving their capability of interacting with the external environment [18]. Accordingly, such adaptations were supposed to take part in the interaction of *B. laterosporus* spore components with the host midgut barrier after ingestion to initiate the pathogenic action [19]. It has been shown that spore ingestion by house fly larvae is followed by a series of events, including the disruption of epithelial microvilli structure, vacuolization, and disorganization of the cytoplasm up to cell lyses [20]. Midgut barrier breaking is followed by bacterial septicemia in the host, where the bacterium can take advantage of the nutrient-rich hemocelic environment where the vegetative cells can proliferate. According to this scenario, the predicted virulence properties of S-layer proteins and their abundant release in the insect body are expected to contribute to the progress of the infection. On the other hand, in this stage, other virulence factors of *B. laterosporus*, such as proteolytic and chitinolytic enzymes, may play a more significant role in the degradation of the host tissues, while others may have a cytotoxic action [13,14].

In general, the main functions associated with bacterial S-layers include adhesion to external surfaces and evasion of the host immune system in the case of pathogens. For example, it has been observed that cell-surface proteins of certain strains within the *Bacillus cereus* group are involved in adhesion and interaction with host tissues and cellular components [21]. Analogously, surface-layer proteins of the honeybee pathogen *Paenibacillus larvae* are involved in the bacterial adhesion to epithelial cells in the larval midgut, representing a preliminary interaction with the host to initiate the pathogenic process [22]. S-layer proteins of pathogenic bacteria have also been observed to play a role in the interaction with the host immune system. Accordingly, these proteins were reported to be involved in the host immune mechanism evasion by the human opportunistic pathogen *Campylobacter fetus*. On the contrary, certain S-layer proteins of probiotic bacteria seem to be responsible for triggering the host immune response [23]. In accordance with these studies, it could be speculated that S-layer proteins of *B. laterosporus* may also intervene directly in the pathogenic process, in combination with other virulence factors. Our study highlighted for the first time the possible implication in the pathogenic process of the S-layer proteins that are abundantly produced and released by vegetative cells. While their specific role in different stages of pathogenesis inside the host remains to be determined, it is likely that in analogy with other species, S-layer proteins contribute to the adaptive abilities of this ubiquitous bacterial species to a wide variety of environments. In fact, in addition to its role as an entomopathogen, *B. laterosporus* has often been reported as a resident of the gut of several animals, playing a role as an enhancer of their immune system and health [24,25]. Accordingly, this bacterium is a common resident of the honeybee body, with potential beneficial effects, being an antagonist of the bee pathogen *P. larvae* [26,27,28]. Finally, it cannot be ruled out that in addition to the production of various antimicrobial compounds, the highly competitive abilities of *B. laterosporus* toward other microorganisms may also take advantage of the biological properties associated with S-layer proteins. Future studies will be needed to define the actual potential of such proteins for exploitation in the different areas where this multivalent bacterium is relevant.

## 4. Materials and Methods

### 4.1. Bacterial Strain and Growth Conditions

The insecticidal strain *B. laterosporus* UNISS 18 (=NCIMB 41419), whose activity against Diptera is well-documented [29], was used in this study. Cultures were routinely conducted in Luria–Bertani (LB) broth at 30 °C, shaking at 180 rpm, according to methods described by Marche et al. [12] to synchronize bacterial growth. Briefly, LB pre-culture was inoculated with an aliquot (1 mL) of heat-activated (80 °C for 10 min) fresh spore suspension, grown to the exponential phase, and used to inoculate T3 sporulation medium. Culture progress was checked in each flask by phase contrast microscopy to harvest different stages of growth and bacterial fractions (vegetative cells and spores) by centrifugation (5000 rpm for 20 min at 4 °C), as needed for bioassays and analyses. Differential centrifugation of the whole sporulated culture was used to separate spores and the above-described protein fraction containing released cell wall multilayers (RWL). This fraction was washed in sterile water (three centrifugation cycles at 15,000 rpm for 20 min at 4 °C) before being used in bioassays, to eliminate possible soluble compounds released by the bacterium during culture in a liquid medium.

### 4.2. Cell Wall Observation by Transmission Electron Microscopy

After being harvested from liquid culture, bacteria were incubated for 24 h at 4 °C in 4% glutaraldehyde (Merck, Darmstadt, Germany) and 4% paraformaldehyde (Merck, Darmstadt, Germany) in cacodylate buffer (0.05 M, pH 7.2) at 4 °C for fixation. Then, cells were washed in the same buffer and suspended in low-melting-point agarose, whose pieces were fixed in 1% osmium tetroxide for 1 h at 4 °C, washed in the buffer, and dehydrated in ethanol, before being embedded in Epon-Araldite. Ultra-thin sections were observed and photographed under a Zeiss EM 109 transmission electron microscope, after treatment with uranyl acetate and lead citrate.

### 4.3. Protein Analyses

Two protocols were used to specifically extract different cell wall layer proteins from bacterial cells harvested from 18 h LB cultures at 30 °C. According to the first protocol [30,31], cells were washed in sterile water before being re-suspended in 1 mL of 2 M guanidine hydrochloride pH 2.5 (Merck, Darmstadt, Germany) and left for 30 min at room temperature. After centrifugation, the supernatant was collected and dialyzed against 100 mM of Tris-Cl pH 8, using SnakeSkin^TM^ Pleated Dialysis tubing, 3500 MWCO (Thermo Fisher Scientific, Waltham, MA, USA). The buffer was refreshed during dialysis after the following time intervals: 1, 1, and 2 h. In the second method [17], harvested cells were washed in 50 mM of Tris-Cl pH 7.5 (Merck, Darmstadt, Germany), and re-suspended in 5 mL of the same buffer containing 5 mM of EDTA (Merck, Darmstadt, Germany), before being incubated at 37 °C for 30 min with slow shaking. After centrifugation at 15,000 rpm for 10 min, cold acetone was added to the supernatant up to a 70% concentration. The suspension was incubated overnight at −20 °C before centrifugation. The pellet was washed in 1 mL of cold acetone (Merck, Darmstadt, Germany) twice, centrifuged, and re-suspended in 250 μL of 50 mM Tris-Cl, pH 7.5, before being incubated at room temperature with shaking for 1 h.

Further protein extractions were conducted on the whole sporulated culture containing lysed sporangia, free spores, and cell wall debris. For this purpose, 48 h liquid cultures in LB were harvested by centrifugation at 15,000 rpm for 10 min at 4 °C and resuspended in ice-cold 0.5 M NaCl. Each suspension was whirl-mixed-sonicated and centrifuged at 13,000 for 5 min. The pellet was resuspended in 1% SDS, 0.01% β-mercaptoethanol (Merck, Darmstadt, Germany), before being boiled for 10 min and re-centrifuged at 13,000 for 10 min. The resulting supernatant was collected for analysis.

One-dimensional gel electrophoresis (1-DE) protein separation analyses were conducted by mixing various protein samples with Laemmli buffer (Merck, Darmstadt, Germany) [32], boiling for 5 min, and running in a 10% SDS-PAGE gel employing a Mini-Protean electrophoresis system (BioRad Laboratories Inc., USA). Gels were digitized after being stained with Coomassie brilliant blue (Merck, Darmstadt, Germany).

Folin phenol reagent (Merck, Darmstadt, Germany) [33] was used for protein concentration determination using bovine serum albumin (Sigma) as a standard.

Identification of major proteins in 10% polyacrylamide gel, extracted from bacterial cell walls, was based on LC MS/MS analyses conducted as described elsewhere [12]. N- terminal sequencing was carried out for a main band (just below 120 kDa) in 10% SDS-PAGE of whole sporulated culture proteins. For this purpose, proteins separated by 1D SDS-PAGE were electroblotted onto polyvinylidene fluoride (PVDF) membrane (Immobilion P, Merck Millipore Corporation, Germany), and the protein band was analyzed by N-terminal Edman sequencing. This protein analysis was carried out by the Protein and Nucleic Acid Chemistry Facility (PNAC) of the Department of Biochemistry (University of Cambridge, UK).

The nucleotide sequences of the identified cell wall proteins were analyzed on the genome of *B. laterosporus* strain UNISS 18, available from NCBI with accession No. MBFH00000000 [34].

### 4.4. Insect Bioassays

The bioactivity of different bacterial fractions was determined through bioassays on *Musca domestica* L. adults provided by the rearing laboratory of the Department of Agricultural Sciences of the University of Sassari (Italy). Each experimental unit consisted of 10 newly emerged flies placed in a transparent plastic jar (7 cm diameter and 12 cm high) with one side covered with gauze to allow ventilation. Flies in a cage were fed a 30% saccharose solution pre-mixed with the bacterial preparation to be assayed or left untreated (control). Food administration was conducted daily by capillary tubes (7.5 μL/fly/day) [35]. The experimental design involved four replicates, and each experiment was repeated three times. The following bacterial preparations were assayed: (i) vegetative cells, (ii) whole sporulated culture with free spores and cell wall remnants, (iii) pure spores, and (iv) a fraction of released cell wall multilayers (RWL). For comparing different bacterial fractions, their concentrations were normalized to 10^9^ cells/spores per mL. The concentration of the RWL fraction used in bioassays corresponded to the amount harvested from a whole sporulated culture with a concentration of 10^9^ spores per mL.

### 4.5. Statistical Analysis

Statistical analyses of processed data were conducted with R software version 4.2.0 [36].

Comparisons between different treatments and the control were performed with one-way ANOVA (factor: treatment), followed by the LSD post-hoc test. 

Over time, the insect survival rate was analyzed by repeated measures ANOVA (proc. mixed), and means were separated by LSMEANS comparison (adjust = Tukey).

The relation between insect mortality and the protein concentration of the RWL fraction was analyzed by linear regression analyses.

## Data Availability

The data that support the findings of this study are available upon reasonable request.
